# International experience of pregnancy outcomes in auto-inflammatory syndromes treated with Interleukin-1 inhibitors

**DOI:** 10.1186/1546-0096-13-S1-O67

**Published:** 2015-09-28

**Authors:** T Youngstein, P Hoffmann, T Lane, R Williams, D Rowczenio, H Ozdogan, S Urgurlu, J Ryan, L Harty, S Riminton, A Headley, J Roesler, N Blank, C Michler, A Simon, P Hawkins, H Lachmann

**Affiliations:** 1 University College London, National Amyloidosis Centre, Division of Medicine, London, UK; 2 National Institute of Health, National Human Genome Research Institute, Bethesda, USA; 3 Cerrahpasa Medical School, University of Istanbul, Division of Rheumatology, Istanbul, Turkey; 4 Cork University Hospital, Department of Rheumatology, Cork, Ireland; 5 Concord Hospital, Department of Immunology, Sydney, Australia; 6 University Hospital Carl Gustav Carus, Department of Paediatric Immunology, Dresden, Germany; 7 University of Heidelberg, Division of Rheumatology, Heidelberg, Germany; 8 University Medical School, Autoinflammation Reference Centre, Teubingen, Germany; 9 Raboud University, General Internal Medicine, Nijmegen, Netherlands

## Introduction

Many patients on anti-IL-1 therapy are unable to stop treatment prior to conception or during pregnancy but little data exist regarding safety. We report the outcomes of 20 IL-1 inhibitor exposed pregnancies in 18 women from 7 countries (including the first data on canakinumab exposed pregnancy) and paternal exposure to anakinra or canakinumab at conception.

## Objectives

To collate data on outcomes of IL-1 inhibitor exposed pregnancies.

## Patients and methods

A standardised data collection sheet was sent to clinicians known to treat SAID. Responses were collated and analyzed retrospectively.

## Results

We received data on 20 pregnancies born to 18 women (Diagnoses: CAPS (11), AOSD (2), TRAPS (2), FMF (2), and Idiopathic Pericarditis (1)).

4 pregnancies were exposed to canakinumab with theoretical exposure up to 18 weeks; 18 were exposed to anakinra (2 were exposed to both agents).

All pregnancies reported were completed. The median gestation at delivery was 38+4 weeks (range 35+1 to 41). APGAR score was >9 in all recorded cases, birth weight range 2.02-3.94 kilos.

Two congenital abnormalities were reported in a single infant; growth hormone deficiency due to ectopic neurohypophysis and unilateral renal agenesis in a boy born to a mother with active AOSD at conception treated with anakinra from 9 weeks gestation.

7 babies were breast fed by mothers receiving anakinra (range 3 - 40 weeks). None have reported infections or developmental abnormalities.

A total of 13 babies were born to 10 fathers treated with either anakinra (5) or canakinumab (8) at conception with no reported abnormalities.

## Conclusion

This is the largest study to date of pregnancy outcomes in parents receiving anti-IL1 therapies. Overall the data are reassuring but numbers remain small.

Based on available data we currently advise preconception planning, with the risks and benefits of therapy individually discussed with potential parents. The data regarding canakinumab are limited and our advice is to switch to the more physiological anakinra, ideally prior to conception. Whilst anakinra is detectable in breast milk the data from 7 infants suggests it has no deleterious effects.

The significance of unilateral renal agenesis in a foetus exposed to anakinra is uncertain given a previous report of fatal bilateral renal agenesis *in utero* of a twin foetus exposed to anakinra (Chang *et al*., Arthritis & Rheumatology 2014). This requires further investigation, and there is a need for the SAIDs community to develop a registry in order to provide the best information for patients.

